# Associations Between Maternal Community Deprivation and Infant DNA Methylation of the SLC6A4 Gene

**DOI:** 10.3389/fpubh.2020.557195

**Published:** 2020-11-27

**Authors:** Kelly DeLano, Alonzo T. Folger, Lili Ding, Hong Ji, Kimberly Yolton, Robert T. Ammerman, Judith B. Van Ginkel, Katherine A. Bowers

**Affiliations:** ^1^Cincinnati Children's Hospital Medical Center, Division of Biostatistics and Epidemiology, Cincinnati, OH, United States; ^2^Xavier University, Department of Mathematics, Cincinnati, OH, United States; ^3^Every Child Succeeds, Cincinnati, OH, United States; ^4^Department of Pediatrics, University of Cincinnati College of Medicine, Cincinnati, OH, United States

**Keywords:** community, deprivation, epigenetics (DNA methylation, histone modifications), poverty & inequality, prenatal

## Abstract

**Introduction:** Poverty is negatively associated with health and developmental outcomes. DNA methylation (DNAm) has been proposed as a mechanism that underlies the association between adversity experienced by mothers in poverty and health and developmental outcomes in their offspring. Previous studies have identified associations between individual-level measures of stress and adversity experienced by a mother during pregnancy and infant DNAm. We hypothesized that independent of individual stresses, a mother's community-level deprivation while she is pregnant may also be associated with DNAm among the genes of her offspring that are related to stress response and/or development.

**Methods:** Pregnant mothers (*N* = 53) completed assessments that measured stress, adversity, and mental health. To evaluate community-level deprivation, mothers' addresses were linked to census-level socioeconomic measures including a composite index of deprivation that combines multiple community-level indicators such as income and highest level of education received. Infant buccal cells were collected at about age 4 weeks to measure DNAm of candidate genes including *NR3C1, SCG5*, and *SLC6A4*, which are associated with the stress response and or social and emotional development. Multivariable models were employed to evaluate the association between maternal community deprivation and infant DNAm of candidate genes.

**Results:** No significant associations were identified between maternal community-level deprivation and the methylation of *NR3C1* or *SCG5*, however, maternal community-level deprivation was significantly associated with higher mean methylation across 8 CpG sites in *SLC6A4*.

**Conclusion:** This study identified an association between community-level measures of deprivation experienced by a mother during pregnancy and DNAm in their offspring. These findings may have implications for understanding how the community context can impact early biology and potential function in the next generation.

## Introduction

The lifestyle and health of a mother during pregnancy can significantly affect offspring health. For example, exposure to maternal depression and anxiety is related to increased behavioral reactivity and cortisol levels in infants and is associated with reduced gray matter in the frontal cortex (1). Prenatal maternal stress is also associated with increased risks of psychopathology; decreased cognitive, linguistic, and play abilities; behavioral problems; and increased heart rate in infants (1, 2). Prenatal depression has been associated with increased cortisol levels in infants and altered neurobehaviors (3). Additional research has begun to uncover the long term effects a mother's pregnancy experience has on the life of the child as he or she grows older (4). In addition to individual maternal adversity and mental health, a mother's community, neighborhood, and social environments are also associated with infant and child development. Poverty during early childhood can lead to structural differences in brain development and related deficits in academic achievement (5, 6).

One proposed mechanism linking the maternal social environment to biologic changes in the offspring is epigenetics. Epigenetics refers to chemical modifications to chromatin, such as the binding of methyl groups to DNA, that regulate genomic transcription and may be sensitive to early environmental signals (4). For example, studies have shown increased exposure to parental stress is associated with changes in DNAm in infants (7). To associate maternal and early adversity to infant DNAm, prior studies have employed both epigenome-wide and candidate gene approaches, with a focus on genes involved in the neuroendocrine response and neurodevelopment (2, 8, 9). Evidence also suggests that differences in DNAm at certain genes is associated with development and behavior (10). However, prior studies have focused primarily on individual-level, rather than community-level adversity and the association with infant DNAm.

Community disadvantage may lead to changes in DNAm that can have functional consequences to cognitive and behavioral health that persist into adulthood. At the community-level, disadvantage can mean increased food insecurity, violence, and housing instability, all factors disruptive to early development (11). Two recent studies support a relationship between community disadvantage and DNAm at genes in the pathways of stress reactivity, inflammation and neurodevelopment (12, 13). These community-level effects appear to be independent of individual socioeconomic factors, suggesting that community deprivation has deleterious effects that extend beyond individual circumstances. The epigenetic response to community disadvantage is linked to structural brain differences in regions that impact executive function and emotional regulation (13).

The purpose of this study was to determine the association between community deprivation and offspring DNAm at loci in the regulatory regions of candidate genes. Three candidate genes were selected based on prior research and associations between maternal stress and mental health in pregnancy and infant DNA methylation. The Nuclear Receptor Subfamily 3, Group C, member 1(*NR3C1*) glucocorticoid receptor has been well studied due to its essential role for modulating the stress response through regulation of the hypothalamic-pituitary-adrenal (HPA) axis. Prior research has shown that methylation of this gene is associated with psychosocial stress and a variety of stress-related disorders (14). These associations have been observed for stress experienced across the life-course. DNA methylation in the Solute Carrier Family 6, Member 5 (*SLC6A4*) serotonin transporter gene has been associated with exposure to adversity such as maternal depression and childhood trauma (15). In addition to associations with maternal adversity, this receptor is essential for socio-emotional and behavioral development and has been shown to be epigenetically modified in people who experience major depressive disorder. Less evidence exists regarding the role of the Secretogranin V (*SCG5*) gene in either a response to stress and adversity or infant development. The *SCG5* gene encodes a chaperone protein that is widespread in neuroendocrine tissues and, notably, high maternal prenatal distress has been associated with lower offspring DNA methylation and *SCG5* gene expression (8). Differential infant DNAm of these candidate genes attributed to a mother's prenatal experience may underlie health consequences to the infant, such as delayed development.

## Materials and Methods

### Study Population

We conducted this analysis within the Pregnancy and Infant Development (PRIDE) Study. Participants from the PRIDE Study were enrolled from the Every Child Succeeds (ECS) early childhood home visiting program in the Greater Cincinnati, Ohio area. The program provides home visiting services to mother-child dyads in seven counties in southwest Ohio and Northern Kentucky. All mothers in the program are low income, single, or have other psychosocial risks. Women who enroll prenatally receive weekly, bi-weekly, or monthly visits depending on their gestational week. After the infant is born, the home visits continue until the child is age 3 years, and include regular developmental screening beginning at age 4 months. The women participating in the PRIDE Study cohort were required to be English speaking, 18 years of age or older, and between 12 and 35 weeks gestation. Eligible participants were referred to the PRIDE Study by ECS home visitors. All procedures of the PRIDE Study were approved by the Institutional Review Board at Cincinnati Children's Hospital, and mothers provided informed consent for their and their infant's participation.

### Study Visits

Two home visits were conducted for the PRIDE Study. The first home visit was conducted prenatally during the second or third trimester of pregnancy and the second home visit was conducted postnatally when the infant was age 3–5 weeks. At the first visit, informed consent was obtained, data on maternal stress, adversity, and social support during childhood and pregnancy were collected, and a hair sample was collected. The purpose of the second home visit was to measure infant neurobehavior and to collect buccal cells from infants for DNAm analysis.

### Directed Acyclic Graph

**Figure 1:**
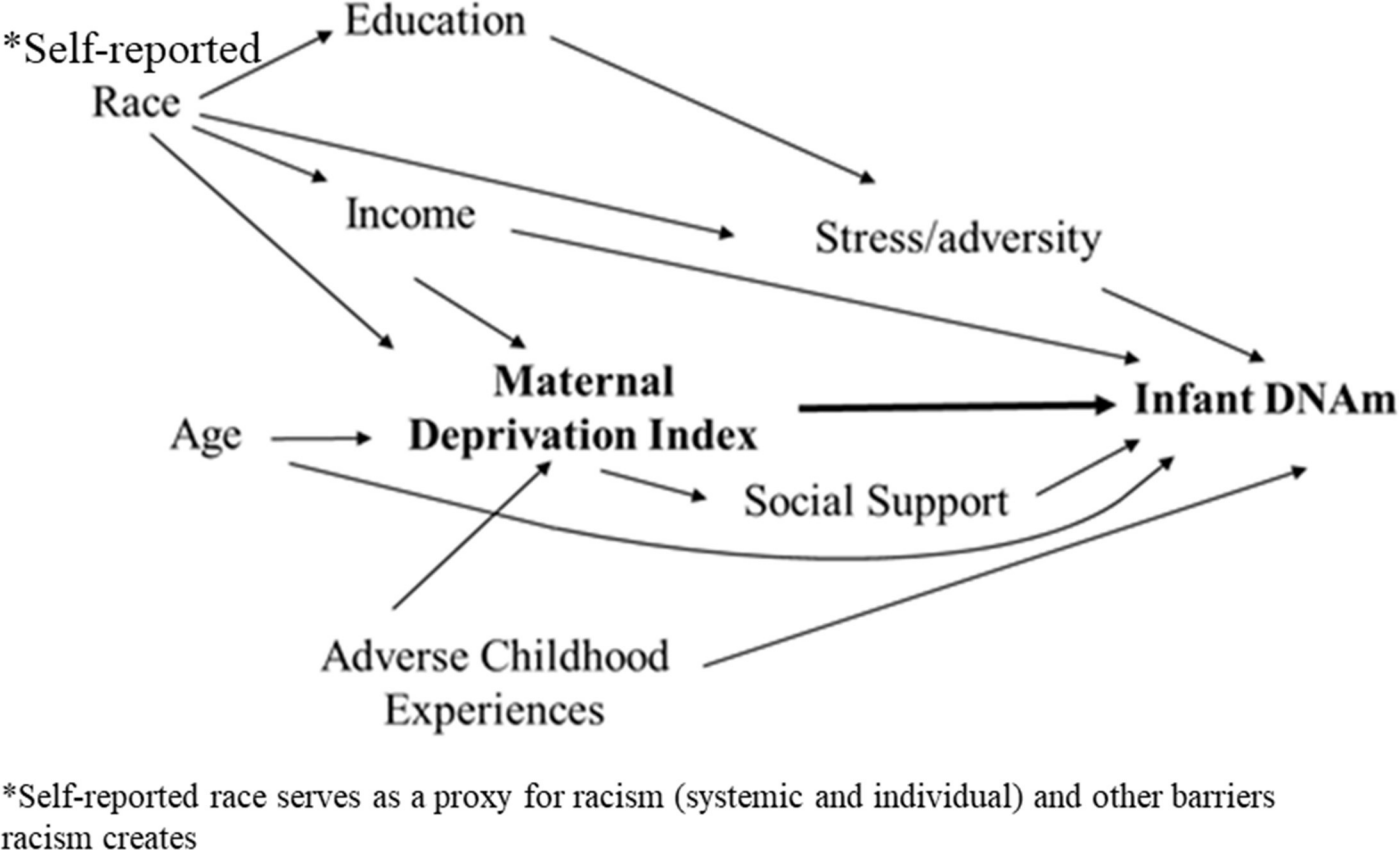
Directed Acyclic Graph displaying the hypothesized association between maternal deprivation index and infant DNA methylation.

Directed Acyclic Graphs (DAGs) are used to display hypothesized relationships and causal pathways. In a DAG, an arrow connecting one variable to another represents causation. Therefore, if there is not an arrow directly connecting two variables, there is not a causal association. DAGs are especially helpful for identifying confounding factors and for reducing biases that may occur when variables are inappropriately added to statistical models (16). We developed a DAG to guide statistical modeling of the association between maternal community deprivation and infant DNA methylation (Figure 1) utilizing available measured covariates including household income, mother's age, self-identified race, and Adverse Childhood Experience Scale (ACE) score, and identified maternal stress and social support as potential mediators.

## Measures

### Deprivation Index

We identified census tracts in which each mother lived based on her address at study enrollment. For each census tract, a deprivation index was calculated. The deprivation index combines community-level indicators including the following factors: fraction of people receiving assisted income, fraction of people who graduated from high school, median income, fraction of people with health insurance, fraction of people living in poverty, and fraction of vacant housing in the community (17, 18). The deprivation index ranges from 0 to 1, where 1 indicates greater deprivation. For the present analyses, deprivation index was evaluated both continuously and dichotomously where a deprivation index above the median (0.32) was designated “high deprivation” and below the median was designated “low deprivation.”

### Additional Individual-Level Measures

Adversity was measured by a variety of self-assessments during the prenatal study visit. The Adverse Childhood Experience Scale (ACE) is a 10-question assessment of physical and emotional abuse and neglect (19). The Edinburgh Postnatal Depression Scale (EPDS) (20) is used to measure maternal depressive symptoms and has been validated for use prenatally (21). The Perceived Stress Scale (PSS) is the most widely used measure of perceived stress and measures the degree to which situations in one's life are appraised as stressful (22). The Interpersonal Support Evaluation List (ISEL) is a 40-item assessment that gives an overall score of support and has four subscales including appraisal, tangible, self-esteem, and belonging (23).

Additional variables included infant sex, maternal age (years) at the time of enrollment, self-reported race (black, white, other), income level (< $25,000, ≥$25,000), high school education (yes/no), and residence distance from major roadways (meters).

### DNA Methylation

DNA was extracted from infant buccal cells collected during the postnatal visit. Pyrosequencing was used to quantify DNAm of the *NR3C1, SLC6A4*, and *SCG5* genes and methylation was measured in Beta-values that indicated percent methylation (Table 1). The Beta-values were converted to M-values with a logit transformation. Cell type heterogeneity was evaluated from an additional sample of infant buccal cells collected at the same time as the DNA and then placed in phosphate-buffered saline. Cells were spun and examined under a microscope to determine the proportion of various cell types. It was found that 99% of the cells were epithelial indicating no need for statistical adjustments for cell type composition.

**Table 1:** Bisulfite pyrosequencing primers.

***NR3C1*** **1F Assay 2** chr5: 143,404,013-143,404,147^a^ (- strand)	
PCR primer forward (5^′^ biotinylated)	GTTGTTATTAGTAGGGGTATTGG
PCR primer reverse	AACCACCCAATTTCTCCAATTTCTTTTC
Pyrosequencing primer	CAACTCCCCCACTCCAAACCC
Targeted CpG sites 1–5	chr5: 143,404,124; 143,404,121; 143,404,114; 143,404,099; 143,404,091
***NR3C1*** **1F Assay 1** chr5: 143,404,011-143,404,097^a^ (- strand)	
PCR primer forward	AGTTTTAGAGTGGGTTTGGAG
PCR primer reverse (5^′^ biotinylated)	AAAACCACCCAATTTCTCCAATTTCTT
Pyrosequencing primer	GAGTGGGTTTGGAGT
Targeted CpG sites 6–10	chr5: 143,404,075; 143,404,073; 143,404,063; 143,404,057; 143,404,043
***SLC6A4*** **Assay** chr17: 30,236,070-30,236,156^a^ (- strand)	
PCR primer forward	GTATTGTTAGGTTTTAGGAAGAAAGAGAGA
PCR primer reverse (5^′^ biotinylated)	AAAAATCCTAACTTTCCTACTCT TTAACTT
Pyrosequencing primer	AAACTACACAAAAAAACAAAT
Targeted CpG sites 1–5	Chr17: 30,236,070; 30,236,087; 30,236,101; 30,236,125; 30,236,156
***SCG5*** **Assay** chr15: 32,934,005-32,934,025^b^ (- strand)	
PCR primer forward	GGGTTGTTTTTAGGTGAGTATAGTTTTGAT
PCR primer reverse	CTCAATACTCCCTTCCCCTTAC
PCR primer-nested forward (same as forward)	GGGTTGTTTTTAGGTGAGTATAGTTTTGAT
PCR primer-nested reverse (5^′^ biotinylated)	AACCTCCACCTCAAAAATTTTAACA
Pyrosequencing primer	GGTGAGTATAGTTTTGATG
Targeted CpG sites 1–4	Chr15: 32,934,005; 32,934,009; 32,934,016; 32,934,025

a *Chromosomal coordinates for PCR and sequencing are based on UCSC Genome Browser Human Dec. 2013 (GRCh38/hg38) Assembly*.

b *Chromosomal coordinates for PCR and sequencing are based on UCSC Genome Browser Human Feb. 2007 (GRCh37/hg19) Assembly*.

### Statistical Analysis

For the present analyses, deprivation index was evaluated both continuously and dichotomously. Participant characteristics were compared between the high and low deprivation groups, comparing means (or medians for variables with non-normal distributions) for continuous variables and frequencies and percentages for categorical variables. Normality was assessed for continuous variables using a Shapiro Wilk Test, and a Welch's two sample *t*-tests was used to identify statistically significant differences between the high and low deprivation groups. When the data were not normally distributed, a Wilcoxon Signed-Rank Test was used. Chi Squared and Fisher's Exact tests identified the statistical associations between categorical variables and high and low deprivation groups. Associations between maternal characteristics, adversity measures, social support, and continuous deprivation index were analyzed using simple linear regression.

A multivariable general linear model was used to examine the association between the deprivation index of the mother and infant DNAm. Guided by the DAG, we adjusted the model for mother's age, household income, mother's self-reported race, and mother's ACE score. A potential interaction between deprivation and infant sex (male/female) was investigated by including a multiplicative interaction term in the multivariable model and using stratification. All statistical analyses were completed using R software. Associations with *p* ≤ 0.05 were considered statistically significant.

## Results

We enrolled 56 participants in this phase of the PRIDE Study. Of these, 53 participants completed both the prenatal and the postnatal study visits. Mothers identified themselves as white (38%), black or other race (62%), and Hispanic (6%). Eighty-seven percent of mothers were employed, 51% had a household income of < $25,000, and 79% had no college experience. The distribution of the deprivation index of the sample was generally higher than the overall distribution of the deprivation index across the Greater Cincinnati area. Sociodemographic variables did not significantly across deprivation groups, except self-reported race (*p* < 0.0001) (Table 2). No individual-level adversity measures were significantly associated with deprivation index, including the EDPS, PSS, and ISEL, administered during home visits.

**Table 2:** Maternal characteristics, adversity measures, and measures of support are summarized with linear regression coefficients for the association between each and a continuous measure of the deprivation index as well as means (standard deviations) for continuous variables and the number (percent) for categorical variables presented by the deprivation index variable split at the median.

	**Deprivation**		
	**β**	**High (≥0.32)**	**Low (<0.32)**
**Maternal characteristics**			
Age, yrs	0.49	22.35 (3.83)	21.19 (2.65)
Self-reported Race^*^			
White	Ref	7 (35%)	13 (65%)
Black/Other	7.85	19 (59%)	13 (41%)
**Hispanic**			
Yes	−7.91	1 (50%)	1 (50%)
No	Ref	25 (50%)	25 (50%)
**Employed**			
Yes	1.08	24 (53%)	21 (47%)
No	Ref	2 (29%)	5 (72%)
**Household income**			
< $25,000	Ref	15 (56%)	12 (44%)
>$25,000	−2.43	11 (48%)	12 (52%)
**Education**			
No college experience	Ref	21 (50%)	21 (50%)
Some college/Bachelor's	−0.69	5 (50%)	5 (50%)
Distance from major roadway, meters	0	2,285 (2,276)	3,366 (4,122)
Adversity Measures			
**ACE scores**			
<3	Ref	18 (51%)	16 (48%)
≥3	0.97	8 (44%)	10 (56%)
**EPDS**			
<10	Ref	14 (45%)	17 (55%)
≥10	1.72	12 (57%)	9 (43%)
**PSS**			
Low	Ref	12 (52%)	11 (48%)
Moderate/High	0.34	14 (50%)	14 (50%)
Cortisol	−0.09	16 (55%)	13 (45%)
**Social support**			
Appraisal	−0.6	22.12 (5.35)	24.77 (5.33)
Tangible	−0.31	21.04 (7.87)	23.35 (4.77)
Self-Esteem	−0.02	21.92 (4.66)	22.35 (4.42)
Belonging	0.04	22.04 (7.00)	22.08 (6.78)

* *No variables were statistically significantly different by levels of deprivations, except for race (P < 0.0001)*.

In an unadjusted model, a mother's deprivation index during pregnancy was significantly associated with infant DNAm of *SLC6A4* (β = 3.31, *p* = 0.02). Adjusting for maternal age, the association remained statistically significant (β = 2.81, *p* = 0.03). Adjusting for household income, race, and the mother's ACE score, the association was no longer statistically significant (β = 2.17, *p* = 0.10) (Table 3). Although there was not a statistically significant interaction (*p* > 0.05), as we were underpowered to identify interactions, in stratified analyses the association between maternal deprivation during pregnancy and methylation of *SLC6A4* was smaller among males (β = 1.87, *p* = 0.39) compared to among females (β = 3.69, *p* = 0.14). There were no statistically significant associations observed between maternal deprivation index and DNAm of *NR3C1* or *SCG5* (β = 3.23 (*p* = 0.76) and β = 3.77 (*p* = 0.37), respectively, in unadjusted models).

**Table 3:** Unadjusted and adjusted models illustrating relationship [beta values (*p*-value)] between maternal community deprivation and mean infant DNAm of *SLC6A4*.

**Variable**	**Unadjusted**	**Adjusted I**	**Adjusted II**
Deprivation index	3.31 (0.023)	2.81 (0.03)	2.17 (0.10)
Maternal age		0.17 (0.06)	0.16 (0.11)
Household income			0.45 (0.61)
Self-reported race			0.24 (0.91)
Mother's ACE score			0.01 (0.55)

## Discussion

We identified an association between mothers' community level deprivation index while she was pregnant and infant DNAm of *SLC6A4* gene. The association remained after adjustment for maternal age. Although the association was attenuated and no longer statistically significant after additional adjustment for household income, race, and mother's ACE score, the effect was still evident; statistical significance may have been impacted by our small sample size. While there was not a significant interaction between deprivation index and infant sex, the effect in females was 2-fold the effect in males suggesting a potential heterogeneity of effects that should be tested in a larger sample. No statistically significant associations were identified for CpG sites in *NR3C1* and *SCG5*.

The *SLC6A4* gene encodes the serotonin transporter (5-HTT), which is essential for the reuptake of the neurotransmitter serotonin, facilitating communication between neurons (9). 5-HTT transports serotonin from the synaptic cleft to the presynaptic neuron (24). Serotonin is a chemical compound that modulates many behavioral and neuropsychological processes, and dysregulation of receptors has been associated with psychiatric disorders (25). Previous research has demonstrated an association between prenatal maternal depression and DNAm of the *SLC6A4* gene (9). Therefore, DNAm and related silencing of 5-HTT expression may disrupt normal neuropsychological processes. Alterations in 5-HTT availability may increase risk for later emotional problems such as depression (26).

The observed association between early adversity in the environment and infant DNAm aligns with several prior studies. Specifically, a 2013 study reported a significant relationship between adversity experienced during infancy and preschool and DNAm in adolescents (7). In addition, another study showed that individuals from at-risk populations who experienced significant stress from their early life experiences had greater DNAm of the *SLC6A4* gene (27). Maternal depression during pregnancy has also been associated with offspring *SLC6A4* DNAm, suggesting a potential impact on longer term emotional development (9).

Our study contributes to fledgling literature that examines the relationship between prenatal community-level deprivation and differential offspring DNAm. In one study, it was shown that an increase in one standard deviation of deprivation was associated with significantly higher DNAm of the *MEG3* gene in infants (28). In two other studies, community disadvantage was linked to significant differences in DNAm at genes controlling stress reactivity, inflammation, and neurodevelopment (12, 13). The current study adds *SLC6A4* to the list of genes involved in stress response, and more broadly, behavioral and neuropsychological processes that may have regulatory differences by community context. This information suggests that the multitude of factors at the community level can have a potent impact on offspring biology with potentially, long-term consequences.

The study had several limitations. First, the sample size precluded detection of anything other than large effects. Therefore, it is possible that other, more modest associations between community deprivation and CpG sites of other genes existed and will be important to examine in larger studies. Second, although several measures were taken regarding the experience and health of the mother before and during pregnancy, many other factors including nutrition and pollution of the environment in which the pregnant mother and infant lived may affect DNAm and were not measured in the present study. These variables could modify the identified association and should be investigated further. We also did not have the power to investigate potential mediation and effect modification of this association.

There were also important strengths to this study. First, the longitudinal study design permitted prenatal assessment of community deprivation characteristics followed by a neonatal measurement of offspring DNAm. Second, the study examined these associations within a low-income, urban cohort that was representative of families who participate in early childhood prevention programs for high-risk families. The study population permits generalization to the large system of early childhood home visiting programs serving families throughout the United States. Although all the mothers had high sociodemographic risk, there was still variation in the index of deprivation enabling estimation of the effect on DNAm. Third, the study uniquely investigated the effect of community context on offspring epigenetic mechanisms. This work provides insight into how community variation, even within lower income communities, can impact early biology. Together, the study design, biologic plausibility of candidate gene selection, and strong effect size after controlling for several covariates, suggest a potential association that warrants replication in larger studies.

We identified a novel association between the deprivation of the maternal community during pregnancy and infant methylation of DNA at the *SLC6A4* gene. Given the role of *SLC6A4* in social and emotional health, DNAm of *SLC6A4* may underlie some of the association between maternal community deprivation and development in infants. Further research is necessary to replicate these findings.

## Data Availability Statement

The raw data supporting the conclusions of this article will be made available by the authors, without undue reservation.

## Ethics Statement

The studies involving human participants were reviewed and approved by Cincinnati Children's Hospital Medical Center. The patients/participants provided their written informed consent to participate in this study.

## Author Contributions

KB and AF conceived and designed the study and supervised the study. KD conceived the statistical approach and preformed the statistical analysis and wrote the manuscript. LD supervised the statistical analyses. KY performed developmental testing on all study visits and critically reviewed analyses. All authors contributed to the article and approved the submitted version.

## Conflict of Interest

The authors declare that the research was conducted in the absence of any commercial or financial relationships that could be construed as a potential conflict of interest.
